# Prisoners of Addictive Cues: Biobehavioral Markers of Overweight and Obese Adults with Food Addiction

**DOI:** 10.3390/nu12113563

**Published:** 2020-11-20

**Authors:** Roni Aviram-Friedman, Lior Kafri, Guy Baz, Uri Alyagon, Abraham Zangen

**Affiliations:** Department of Life Sciences and the Zlotowski Center for Neuroscience, Ben-Gurion University, Be’er Sheva 8410501, Israel; lior363@gmail.com (L.K.); guy.s.baz@gmail.com (G.B.); uri.alyagon@gmail.com (U.A.)

**Keywords:** food addiction, obesity, brain asymmetry, event-related potentials, food stroop, attention bias, cue responsivity

## Abstract

Obesity is associated with food and eating addiction (FA), but the biobehavioral markers of this condition are poorly understood. To characterize FA, we recruited 18 healthy controls and overweight/obese adults with (*n* = 31) and without (*n* = 17) FA (H-C, FAOB, NFAOB, respectively) to assess alpha brain asymmetry at rest using electroencephalogram; event-related potentials following exposure to high-calorie food (HCF), low-calorie food (LCF), and nonfood (NF) images in a Stroop paradigm; reaction time reflective of the Stroop bias; and symptoms of depression and disordered eating behavior. The FAOB group had the greatest emotional and uncontrollable eating, depressive, and binge-eating symptoms. The FAOB group displayed lower resting left alpha brain asymmetry than that of the NFAOB group. Differently from the other groups, the FAOB group presented attenuated Stroop bias following exposure to HCF relative to NF images, as well as a lower late positive potential component (LPPb; 450–495 ms) in both frontal and occipital regions. In the total cohort, a correlation was found between the Stroop bias and the LPPb amplitude. These results point to biobehavioral hypervigilance in response to addictive food triggers in overweight/obese adults with FA. This resembles other addictive disorders but is absent in overweight/obesity without FA.

## 1. Introduction

Obesity is a growing public health condition associated with significant co-morbidities, including diabetes, heart disease, cancer, and depression [1,2]. Consequently, it carries an enormous economic and public health burden [3]. Traditional remedies to reduce the prevalence of overweight and obesity, such as diet and physical activity, are often successful, but most post-obese individuals fail to maintain a healthy body weight in the long run [4]. In the past decade, the clinical construct of addiction to food and eating (i.e., food addiction; FA) has been suggested to explain why some obese individuals are resistant to conventional weight management regimens. The concept of addiction to food is not under a consensus though, and it is not yet recognized in the Diagnostic and Statistical Manual of Mental Disorders (DSM). Proponents of FA posit that there are psychophysiological commonalities between obesity and substance or behavioral addiction [5], while opponents claim that this construct is highly related to binge-eating disorder (BED) [6,7], and that the term "addiction" is inappropriate since food is a legitimate necessity, not a substance individuals can abstain from [8,9]. Nevertheless, eating behavior commonly seen in individuals with overweight and obesity may resemble symptoms of substance or behavioral addiction, as specified in the DSM. This has led researchers to propose FA, by extrapolating criteria for addiction diagnosis in the DSM and translating them to the food and eating behavior domain [10]. These symptoms include frequent and excessive cravings for rewarding food [11], associated with an urgency to relieve stress and negative affect; hypersensitivity to external cues signaling the rewarding food [12]; impulsivity [13] and disinhibition of eating restraint in response to the cues [14]; recurrent overeating past the point of satiety [15], and reduced inhibitory control over eating [16], despite being conscious to the adverse consequences of the behavior [17]. Individuals with these symptoms may experience binge-eating episodes, but many do not satisfy the diagnostic criteria for BED, such as eating binges that occur in a 2-h time window [18]. In fact, in a sample of obese treatment seeking BED patients, only a subset of them met FA criteria [19,20]. Therefore, addictive-like eating symptoms may accelerate chronic obesity rates, above and beyond the incidence of BED.

The psychobiological mechanisms of recurrent overeating in overweight and obesity are partially characterized. A decade of research in this area has been channeled into several theories to explain excessive overeating for reward [21]. The incentive sensitization theory, for example, posits that by repeatedly overconsuming rewarding food, regardless of metabolic hunger, a conditioned food-reward response is formed. The rewarding food is commonly hyperpalatable, high in calories, ultra-processed fat, sugar, and/or salt, and it is considered to have an addictive potential [22,23]. With excessive repetition of this behavior, the conditioned response transfers from a consummatory to an anticipatory food reward (i.e., the reward associated with anticipating the consumption of this food) [24]. In this way, a food cue, such as its olfactory or visual properties, is paired with an anticipatory reward in a conditioned learning mechanism, which stems from neurobiological changes in the mesolimbic dopaminergic system [12]. The anticipatory reward from consuming hyperpalatable food can elicit intense cravings in response to the relevant cue [25]. These food cravings are associated attention bias (AB), or the allocation of attentional resources to the relevant cue. Indeed, multiple studies have confirmed the heightened allocation of attentional systems to food cues in obese, compared with lean individuals [26,27,28]. 

Another theory that may explain recurrent overeating in obesity is the inhibitory control deficit theory. This theory emphasizes that individuals keep overeating due to dysfunctional activity in brain regions involved in higher-order control functions, which are under the command of inhibitory control regions in the prefrontal cortex (PFC). According to this theory, a dysfunctional PFC activity may accelerate repeated overconsumption of highly rewarding food, even in the absence of metabolic hunger, due to reduced inhibitory control over eating [15]. 

The two neurocognitive theories described herein are in line with the approach-avoidance motivational direction theory, according to which individuals with left-sided brain prefrontal asymmetry (i.e., greater left, compared with right, PFC activity) are more likely to be responsive to reward, and/or seek out experiences generating a reward [29]. Left-brain asymmetry is also associated with impaired ability to avoid (or inhibit) behaviors generating undesirable consequences. Differently, individuals with right-sided brain prefrontal asymmetry (i.e., greater right, compared with left, PFC activity) are more likely to avoid experiences generating punishment or disgust, and/or keep away from those generating a reward. 

In line with the asymmetry hypothesis of obesity [30], a growing body of literature suggests that left-brain asymmetry may be a neurocognitive characteristic of uncontrollable hedonic overeating (i.e., the overconsumption of highly rewarding food in the absence of hunger), and impaired inhibitory control over food consumption [31,32]. There is evidence in females ranging from lean to obese for the presence of left PFC asymmetry as a possible mediator of the association between AB to highly rewarding food and a high BMI [33]. Moreover, participants with obesity and BED, compared with obesity only, display greater left-hemispheric regional cerebral blood flow in response to rewarding food [34]. These studies support the hypothesis that left-brain asymmetry is a neuronal mechanism that preserves aberrant eating behavior in obesity and BED, but no study to date examined this neurobiological feature directly in FA. 

The neurocognitive features of heightened attentional sensitivity to an addictive rewarding cue, coupled with inhibitory control deficits over the addictive behavior, and left-brain asymmetry traits, are commonly seen in Substance Use Disorder (SUD) and behavioral addiction [35,36,37]. Therefore, there is some support for similarities between FA and other types of addiction. In an fMRI study of participants ranging from lean to obese, a correlation between FA scores and increased reward circuitry reactivity was found in the Anterior Cingulate Cortex (ACC), medial orbitofrontal cortex (OFC), and amygdala during anticipation of chocolate milkshake consumption [38]. This was observed jointly with reduced inhibitory control activation in the dorsolateral prefrontal cortex (DLPFC) and the caudate in response to the actual consumption of the food, when comparing participants with high vs. low FA scores. Similar neuronal alterations have been identified in classical addiction, in anticipation and the actual intake of the substance [39,40,41,42]. 

Previous studies have identified reward responsiveness and AB to highly rewarding food cues in FA. For example, reward-responsive eating mediated the relationship between elevated dopamine signaling and FA scores [43]. Moreover, in female overweight adults with FA, AB to rewarding food was identified in an eye-tracking paradigm following a negative mood induction, but the neurobiological mechanisms have not been studied [44]. Therefore, no study thus far examined brain asymmetry and neuronal correlates of AB to highly rewarding food in FA, to empirically test these aspects of neurocognition. Moreover, no study to date examined the specific psychobiological distinction between overweight/obesity with FA relative to overweight/obesity without FA. 

To address the knowledge gap in the FA literature, we measured brain asymmetry at rest, cue-reactivity to images of rewarding food in a Stroop Task while measuring EEG, and several psycho-behavioral parameters, including eating behavior (emotional, uncontrollable, restraint, and binge-eating behavior), and depressive symptoms. We compared these parameters between overweight/obese adults with and without FA (FAOB and NFAOB, respectively), as well as lean controls. We hypothesized that the FAOB group, compared with the other two groups, shows greater left-brain asymmetry at rest and AB to food cues, along with heightened evoked electrophysiological responses, in the Stroop task. We also hypothesized greater depressive, uncontrollable and emotional eating, and binge-eating symptoms in the FAOB group, compared with the other two groups. 

## 2. Materials and Methods

### 2.1. Participants

As part of a larger clinical trial aiming to test the effect of repetitive transcranial magnetic stimulation )TMS( on FA and obesity, we recruited 66 adults (ages 18–65), 48 of whom were overweight and obese and 18 were healthy controls (H-C) (body mass index (BMI) >= 28 and 19 ≤ BMI ≤ 25, respectively; see Table 1 for age and gender distribution). Participants were recruited by ads and assessed for several demographic parameters, including BMI and age, using a short screening questionnaire. Before recruitment, participants were also assessed for FA with the Yale Food Addiction Scale (YFAS) [45], of which details can be found in Section 2.3. Psycho-Behavioral Questionnaires below. To be recruited for the FAOB group, participants had to meet three or more YFAS symptoms and score positive on the YFAS clinical distress questions. All obese or overweight participants who did not get an FA diagnosis on the YFAS (i.e., they had either lower than three symptoms, or scored negative on the clinical distress questions) were recruited to the non-addicted overweight and obese group (NFAOB). The H-C comparison group had to have a healthy BMI and show no YFAS FA diagnosis. Therefore, the study included three groups of participants: overweight and obese with FA (FAOB, *n* = 31), overweight and obese without FA (NFAOB, *n* = 17), and healthy controls (H-C, *n* = 18).

Potential participants were required to have no conventional weight loss attempt currently or in the past three months. As part of the screening, all participants filled out a short medical assessment questionnaire to ensure adherence to the inclusion/exclusion criteria of the TMS trial. Exclusion criteria included a cognitive or functional disability diagnosed within the past year; starting or changing a psychotropic prescription in the past three months; substance abuse, current or in the past 12 months; known or suspected pregnancy or lactation; and practicing veganism. Participants signed an informed consent approved by the Soroka Medical Center in Beer-Sheva, Israel.

### 2.2. General Procedures

**Figure A1:**
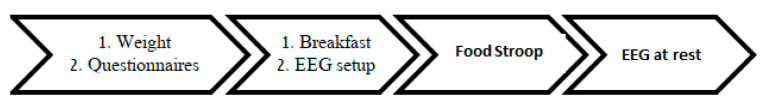
Procedures’ timeline.

The procedures’ timeline is shown in Figure A1. In the current study, we aimed to examine AB to rewarding food cues in the absence of physiological hunger. This is based on evidence pointing to the interaction between physiological hunger and food reward, cravings, and attention bias to food cues [28,46,47,48,49]. We therefore asked the participants to follow preparatory guidelines before arriving at the lab. The guidelines included a dietary menu to follow 24-h before they arrive at the lab. Participants arrived between 9 a.m.–12 p.m. on the day of the study, following an overnight fast (starting at 8 p.m. the night before), except for water or unsweetened hot beverages [50]. Adherence to these guidelines was assessed with a nutritionist upon arrival to the study. At the lab, participants’ weight was measured with a Charder scale (MS4900), and they filled out several questionnaires about their eating behavior (see Section 2.3. Psycho-Behavioral Questionnaires below). Thereafter, they were provided with a standardized breakfast composed of bland food, totaling 640 calories, 43 g of protein, and 27 g of fat. The purpose of the dietary preparation was to standardize hunger and control for metabolic variations between the participants, and by providing the bland breakfast we aimed to intensify their cravings for highly rewarding food cues [47,49,51]. Following breakfast, participants were escorted to a room where EEG recordings and a Food Stroop task were conducted (see below). 

### 2.3. Psycho-Behavioral Questionnaires

#### 2.3.1. YFAS

This is a 25-item instrument asking about eating highly rewarding food, and it provides two types of score: a symptom count (between 1–7) and a clinical significance score (either 0 or 1) that pertains to clinical distress associated with the symptoms experienced. The symptom count is based on the seven substance dependence criteria in the diagnostic and statistical manual of mental disorders, Fifth Edition (DSV-V), where each criterion is measured with item questions containing frequency (i.e., ranging from ‘never’ to ‘four or more times a week, or daily’) or a dichotomous (i.e., ‘yes’ or ‘no’) scoring. For each substance dependence criterion, item scores are added up and transformed to a dichotomous score (i.e., ‘0’ or ‘1’), to reflect if the criterion has been met. All criteria scores are then added up to provide an overall symptom score between 0–7 (see Table A1 for example of items and scoring of the YFAS). 

The clinical significance score pertains to impairment or distress associated with the problematic eating symptoms. This score is calculated based on two questions with five response criteria, ranging from ‘never’ to ‘4 or more times a week, or daily’. Here too, item scores are added up and transformed to a dichotomous score of either ‘0’ or ‘1’, reflecting the final clinical significance score. Food addiction can be ‘diagnosed’ when at least three symptoms, plus the criterion of a clinically significant impairment or distress, are met. This instrument is the most frequently used tool to assess FA in research. It has good psychometric properties (Cronbach’s alpha: 0.84) [52], and it can reliably distinguish between individuals showing symptoms of addictive eating and those who do not [10]. 

#### 2.3.2. Three-Factor Eating Questionnaire (TFEQ) 

Originally a 51-item self-report inventory designed to assess three aspects of eating behavior in obesity [53], the TFEQ has recently been revised to a more concise 18-item instrument with improved psychometric properties [54]. The TFEQ has three subscales: 1. CR (the conscious restriction of food intake to control body weight or to promote weight loss); 2. UE (uncontrollable eating; the tendency to eat more than usual due to loss of control over food intake); and 3. EE (emotional eating; overeating during dysphoric mood states). The Cronbach’s alpha of the TFEQ is 0.79, 0.85, and 0.87 for the three subscales, respectively [54].

#### 2.3.3. Binge Eating (BE) Symptoms 

To assess for BE symptoms, we used the Eating Disorders Examination Questionnaire with Instructions (EDE-Q-I) [55], which is a short questionnaire that can accurately assess the frequency of BE symptoms [56]. This instrument has shown comparable to the gold standard, the EDE interview, in assessing BE symptom frequency [56] (see Appendix B for the specific questions used in the EDE-Q-I to assess BE symptoms). 

#### 2.3.4. Beck Depression Inventory (BDI) 

The BDI is a well-established and highly reliable (Cronbach’s alpha: 0.87) self-assessment questionnaire of depressive symptoms [57]. It contains twenty-one questions, each having a 4-point Likert scale, ranging from 0 (no symptoms) to 3 (severe symptoms). Scores on the BDI indicate mild, moderate, severe, or no depression (represented by scores of 14–19, 20–28, 20–63, and 0–13, respectively) [58]. 

#### 2.3.5. Positive Affectivity Negative Affectivity Schedule (PANAS) 

The PANAS is a self-rating measure of positive affect (PA; 10 items) and negative affect (NA; 10 items), that reflects transitory mood states [59]. This scale has good internal consistency reliabilities (Cronbach’s alpha of 0.88 for the PA and 0.87 for the NA) [60]. Past research has shown the potential influence of affective states on cognitive function in obesity [61]. Therefore, we administered the PANAS following breakfast consumption, to control for the potential effect of affective variability on AB in the Stroop task (see Statistics below).

#### 2.3.6. Visual Analogue Scale (VAS)-hunger

We administered a Visual Analogue Scale of hunger and fullness (VAS-hunger) before the Food Stroop task, to control for a possible effect of variations in hunger and/or cravings for highly rewarding food on attention bias in the Stroop task [62] (see Statistics below). The VAS-hunger is a 7-point Likert scale ranging from 1 (“extremely hungry”) to 7 (“extremely full”), and participants are asked to circle what best describes the way they feel at the moment of measurement.

### 2.4. The Food Stroop Task

The Stroop task is a reliable and widely used neurocognitive tool to assess AB*,* including in addiction research [63]. We administered a Food Stroop task using the E-Prime software (Psychology Software Tools, Inc., Sharpsburg, PA, USA) on a 17’’ computer screen, adjacent to a keyboard with four keys denoted using stickers with different colors (red, green, yellow, and blue; color-key mapping was counterbalanced between the participants). The Food Stroop is a version of the combi-Stroop test [64,65], in which food images precede the Stroop stimuli, to test their influence on participants’ emotional attention. Food images have been commonly used in appetite [66], obesity [67,68], and addiction [63] research. The food images are considered more potent than words [69,70], since they can elicit cravings for food in a similar manner to real food exposure, capturing attention, saliency, and reward [69]. 

**Figure 1:**
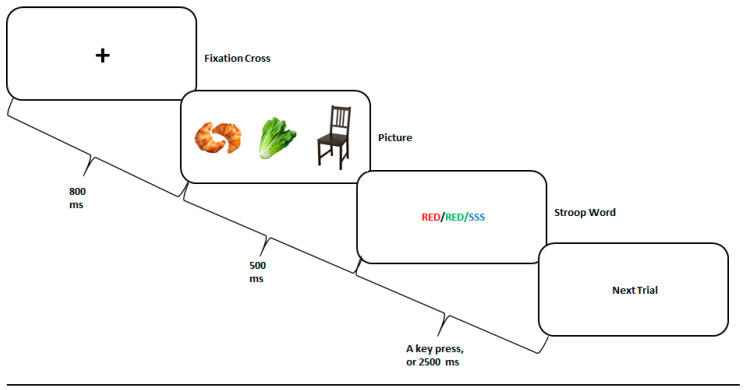
The Food Stroop task experimental paradigm. A fixation cross was presented for 800 ms, followed by a high-calorie food, low-calorie food, or nonfood picture presented for 500 ms, and by a congruent, incongruent, or neutral Stroop word presented for 2500 ms, or until the participant responded. The next trial randomly began 1100–1900 ms thereafter.

The Food Stroop paradigm is illustrated in Figure 1. Each trial started with a fixation cross presented for 800 ms, followed by a food or nonfood image presented for 500 ms. Thereafter, the Stroop word was presented for a maximal duration of 2500 ms, or until the participant responded. The inter-trial interval was randomly set between 1100–1900 ms. 

There were three picture categories: high-calorie food (HCF), low-calorie food (LCF), and nonfood (NF) items, such as furniture. The pictures were retrieved from a freely accessible, previously validated, database [71] and controlled for size, color, and shade. For vegetarian participants, we omitted pictures containing meat and replaced them with vegetarian dishes.

The pictures were followed by either one of the three Stroop conditions, randomly presented. In the three conditions, the meaning of the Stroop word (i.e., “RED”, “GREEN”, “YELLOW”, or “BLUE”) was either congruent, incongruent, or neutral with respect to its color. For example, for the congruent condition, the word “RED” was painted in red; for the incongruent condition, the words “RED” was painted in green, yellow, or blue; and for the neutral condition, a neutral word was presented in any one of the four colors available. The task included 24 practice trials with no pictures presented, followed by 3 blocks of 180 trials each, with a one-minute break in-between. This sums to a total of 540 trials, sixty for each combination of picture typ, and Stroop condition [i.e., (3 picture types × 3 Stroop conditions) × 60]. Overall, 180 pictures were presented, each shown once with each Stroop condition. Participants were instructed to press as quickly and accurately as possible, on the key associated with the color of the word, while ignoring the word’s meaning. 

Mean color-naming latencies (i.e., reaction time, RT) were calculated, excluding error-response trials or ones with RT > 1200 ms. Prior to the beginning of the Stroop task, the VAS-hunger was administered. 

### 2.5. Electrophysiology (EEG) Procedures

EEG was recorded using a 64 electrode Wave guard cap (ANT neuro, Enschede, The Netherlands) and a TMS compatible EEG amplifier (TMSi, Oldenzaal, The Netherlands) referenced to the Cz electrode. Data were acquired using ASA™ version 4.7.3. Impedance was kept below 5 kOhm, and POz was determined as the ground electrode. The recording frequency was set to 2048 Hz and digitized with a 24-bit AD converter. Off-line data processing was conducted using EEGlab 14.0.0 [72] and the FieldTrip toolbox in Matlab (version R2018a; MathWorks Inc., Natick, MA, USA) [73,74], by a trained researcher.

EEG was recorded over a 5-min resting period with lights dimmed, during which the participants were instructed to keep their eyes closed while remaining alert [73,75]. The first 30 and the last 10 s of the resting state EEG data were removed to prevent state transitional influence. The data were filtered using a 1 Hz high-pass and 45–55 Hz notch FIR filters and then epoched into segments of 2 s each. Noisy channels and epochs were detected using an automatic procedure, which was followed by both manual review and rejection, performed by a trained researcher (mean ± SD of epochs and channels rejected in accordance: 14.65 ± 6.02 and 1 ± 0). The criteria for rejection were an absolute amplitude threshold of ±150 µV and an improbability threshold of 3 standard deviations above the mean of the entropy value. An infomax Independent Components Analysis (ICA) was conducted; eye blinks and movements were manually removed (2.3 ± 0.94 components), and a second automatic and manual scan assured no residual noisy segments were left (6.33 ± 7.25 epochs removed). Next, rejected channels were interpolated using spline interpolation; data were re-referenced to the average and transformed to the frequency domain using a fast Fourier transform (1–100 Hz; frequency resolution of 0.125 Hz). Finally, alpha power (8–12 HZ) was extracted for each electrode, and brain asymmetry scores were calculated as the decibel-transformed ratio in alpha power between each left electrode and its homologues right electrode (midline electrodes excluded), using the formula below, previously described [75]:

Left alpha brain asymmetry = 10 × log_10_(*left electrode’s alpha power*) − 10log_10_(*contralateral homologous electrode’s alpha power*) (1)

EEG data collected during the food Stroop task were segmented around the appearance of the food/nonfood image (regardless of Stroop condition), starting at 500 ms before, and ending 1000 ms after image appearance, and then baseline corrected. The data were preprocessed similarly to the resting state EEG analysis, with a manual rejection of noisy epochs (8.19 ± 9.36). Then, an ICA was performed on all recordings (5.05 ± 2.73 components rejected), with additional detection of noisy epochs manually (17.13 ± 17.9) and automatically (31.88 ± 11.73), leaving an average of 161.15 ± 11.31 epochs per food condition. The data were then analyzed in the time domain, as Event-Related Potentials (ERP), in response to a food-specific stimulus. 

Missing behavioral data were removed from the analysis. Table A2 details the number of participants included in the analysis of each variable studied. 

### 2.6. Statistics

All statistical inference tests were performed using two-tailed tests requiring an a-priory alpha level of 5%. Participants’ demographic and clinical data were analyzed using descriptive statistics and a 1-way ANOVA, with the group as a between-subject factor. In the food Stroop task, to capture the maximal emotional and motivational attention elicited by the food cues [63], we focused on the differential effect of HCF and NF images on participants’ performance in the task. Therefore, the Stroop bias score (incongruent-congruent) was computed and analyzed using a 2-way mixed model ANOVA (STATISTICA 13; TIBCO Soft Inc., Seattle, WA, USA) design, with image type (HCF and NF) as a within-subjects factor and the group (FAOB, NFAOB, and H-C) as a between-subjects factor. Post-hoc significance tests were Bonferroni corrected. When analyzing the Stroop bias, we controlled for PANAS as well as for VAS-hunger, based on previous addiction Stroop studies [28,40,44], demonstrating the necessity of controlling for variations between participants in hunger and transitory mood states. 

All EEG data were analyzed using nonparametric permutation tests (Monte Carlo method), implemented via the FieldTrip toolbox. This method samples the data repeatedly and randomly (10,000 iterations) to evaluate the characteristics of the sample’s distribution under the null hypothesis, obviating the need for prior assumptions concerning its normality. 

Brain asymmetry power scores were first averaged for three regions of interest (ROI), including frontal (F5, F3, FC5, and FC3), parietal (CP5 and CP3), and occipital (PO5, PO3, and PO7) electrodes. This method was determined based on previous brain asymmetry research in healthy individuals [76] and in the eating behavior domain [77], depicting these ROI and pointing to their validity in studying brain asymmetry. Next, group differences were tested using permutation tests following the ANOVA logic, i.e., pair-wise post-hoc contrasts were tested only if differences between the three groups were first found significant in one of the ROI [73].

**Figure A2:**
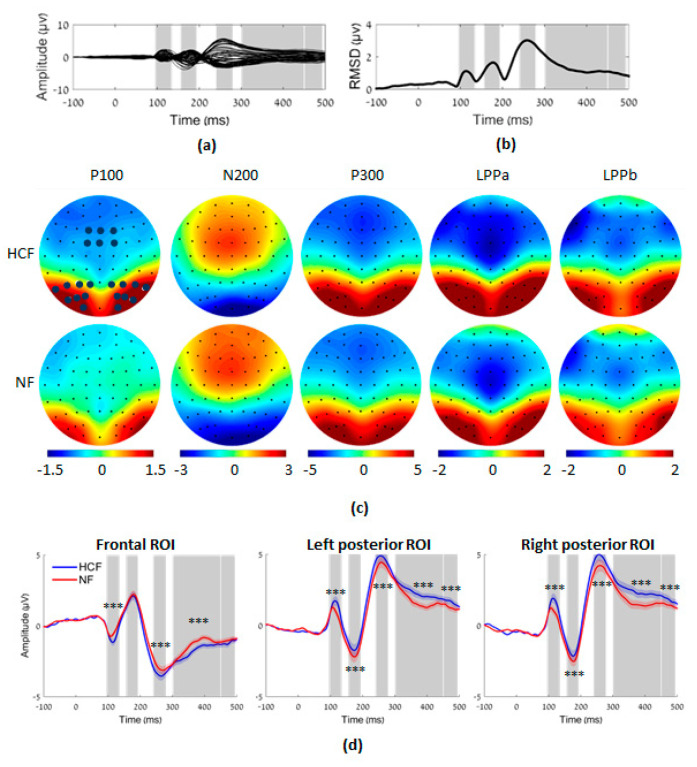
Event-related components in response to food (HCF) and nonfood (NF) images. Data is averaged for all participants together, regardless of group affiliation. (**a**) A butterfly plot of potential curves that include all electrodes. (**b**) Global mean field potential (GMFP) computed sample-wise as the root mean squared deviation (RMSD) from the mean amplitude of all electrodes. The different event-related components and time windows of interest (marked gray) were defined according to the peaks in GMFP and are in line with the literature [65,79]; from left to right: P100 (95–135 ms), N200 (155–195 ms), P300 (240–280 ms), and the Late Positive Potential (LPP) subdivided into LPPa (300–450 ms) and LPPb (450 495). (**c**) Topographic plots of the mean potentials during the different components following HCF and NF images. The frontal, right posterior, and left posterior regions of interest (electrodes marked in dark blue) were defined to capture differences in brain potentials induced by the food images. (**d**) Event-related potential curves in response to HCF and NF images. *** *p* ≤ 0.001. HCF: high-calorie food. NF: nonfood.

ERP components related to the processing of the food images during the food Stroop task were defined prior to the statistical analysis and regardless of group affiliation, using Global Mean Field Power analysis (see Figure A2) and in line with earlier literature [65,78]. We focused our investigation on the Late Positive Potential (LPP) component for two reasons: 1. It reflects the emotional processing of arousing affective pictures [79,80]. 2. It was the only component to show an association with the attention bias induced by the food cues (Figure 5). Note that if not interrupted by another stimulus (i.e., the next trial), the LPP may last a few seconds [79,80]. In contrast, in the current paradigm, this ERP is decreased in preparation for the Stroop word; thus, we subdivided this component into LPPa (300–450 ms) and LPPb (450–495 ms), to better align with differential motivational processes. Three regions of interest (ROI) were defined to capture the anterior and posterior parts of the LPP: frontal (electrodes: F1, Fz, F2, FC1, FCz, FC2), right posterior (P2, P4, P6, P8, PO4, PO6, PO8, O2), and left posterior (P1, P3, P5, P7, PO3, PO5, PO7, O1). Next, mean amplitude differences between HCF and NF were computed for the LPPa and LPPb and subjected to permutation tests as described above.

## 3. Results

### 3.1. Participants’ Demographics and Clinical Data 

Detailed demographic and clinical data of the three groups are shown in Table 1. The groups did not differ in age, gender, or education level. As expected, the three groups differed in FA symptoms; this difference stayed significant when controlling for BE symptoms. As expected, the H-C differed in BMI from both FAOB and NFAOB groups. The three groups differed in their emotional and uncontrollable eating symptoms (TFEQ-EE and TFEQ-UE, respectively), while the FAOB group showed the greatest degree of symptoms. The NFAOB and H-C groups did not differ in depressive or binge-eating symptoms (BDI and BE, respectively), but both groups differed from the FAOB group, who showed the highest level of symptoms. Lastly, the three groups did not differ in cognitive restraint related to food (TFEQ-CR), nor in their PANAS or VAS-hunger scores, but the FAOB group compared with the other two groups, showed a trend for greater hunger (i.e., lower scores on the scale) on the morning of the study.

### 3.2. Hemispheric Brain Asymmetry 

**Figure 2:**
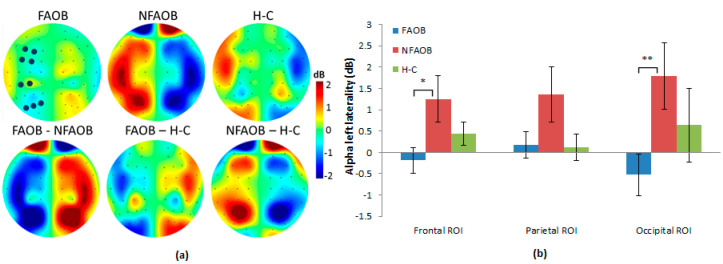
Group differences in brain alpha asymmetry. (**a**) Topographic plots of the mean group left alpha asymmetry power (upper row) and the group contrasts (lower row), for the FAOB, NFAOB, and H-C groups. Note that the maps are based on subtraction of alpha power between symmetric electrode pairs and are thus left/right mirrored. (**b**) Mean and SEM of left alpha asymmetry power for each group in the frontal, parietal and occipital ROI. Electrodes included in each ROI are marked in dark blue in 2a. *p ≤ 0.05; **p ≤ 0.01. FAOB: overweight and obese with food addiction. NFAOB: overweight and obese without food addiction. H-C: healthy controls. SEM: standard error of mean. ROI: region of interest.

**Figure A3:**
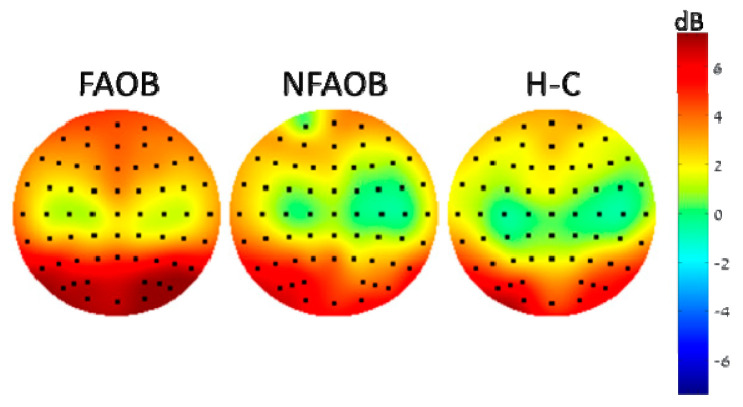
Topographical power maps of the raw resting state EEG activity. Topographical maps of the average resting state power in the experimental groups. No group differences were found using a nonparametric F test in any of the electrodes.

No differences in the raw EEG power were found between the groups (see Figure A3). Non-parametrical permutation tests based on F statistics indicated that brain alpha asymmetry scores differed between the three groups in the frontal (F = 3.45 _(*n* = 49)_, *p* = 0.03) but not in the parietal (F = 2.33 _(*n* = 49)_, *p* = 0.09) or the occipital ROI (F = 2.52 _(*n* = 49)_, *p* = 0.08; (Figure 2)). Post-hoc contrasts indicated that the FAOB group showed lower left alpha brain asymmetry scores (i.e., lower left alpha compared with the right alpha), differently from the NFAOB group, in the frontal and occipital ROI (frontal (*t* = 2.36 _(*n* = 33)_, *p* = 0.02), parietal (t = 1.68 _(*n* = 33)_, *p* = 0.11), and occipital (F = 2.54 _(*n* = 33)_, *p* = 0.01)), but neither of the overweight and obese groups statistically differed from the H-C group. Since the alpha power is a brain frequency inversely related to local brain activity [81], the lower alpha brain asymmetry means greater left, compared with the right, hemispheric dominance in the FAOB group compared with the NFAOB group. Note that nonparametrical tests do not use F or *t* tables; hence, the sample size is detailed rather than the degrees of freedom. Considering previous research [82], we tested for all the participants together the correlation between left alpha brain asymmetry and gender, which was not significant (*r* = 0.19 *p* = 0.15). Therefore, we did not use gender as a covariate in our electrophysiological analyses.

### 3.3. The Effect of Food Cues on Attention Bias and Brain Potentials 

**Figure 3:**
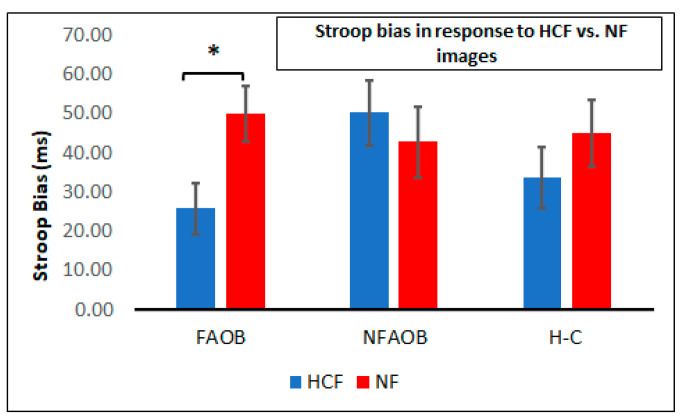
Attention bias following food and nonfood cues. A graphical depiction of mean and SEM of the Stroop bias following HCF and NF images in the FAOB, NFAOB, and H-C participants. * *p* < 0.05. SEM: standard error of mean. HCF: high-calorie food. NF: nonfood. FAOB: overweight and obese with food addiction. NFAOB: overweight and obese without food addiction. H-C: healthy controls.

ANOVA of the Stroop attention bias in response to food cues in the Food Stroop task revealed a significant 2-way group * image type interaction (F _(2,56)_ = 3.46, *p* = 0.04) (Figure 3). This interaction was statistically significant when controlling for VAS-hunger and PANAS scores (mean scores: 6.62 and 20.1, respectively). Post-hoc tests indicated a significant reduction of the Stroop bias following HCF compared to the NF image, which was observed in the FAOB but not in the other two groups (*p* = 0.05; Figure 3). 

**Figure 4:**
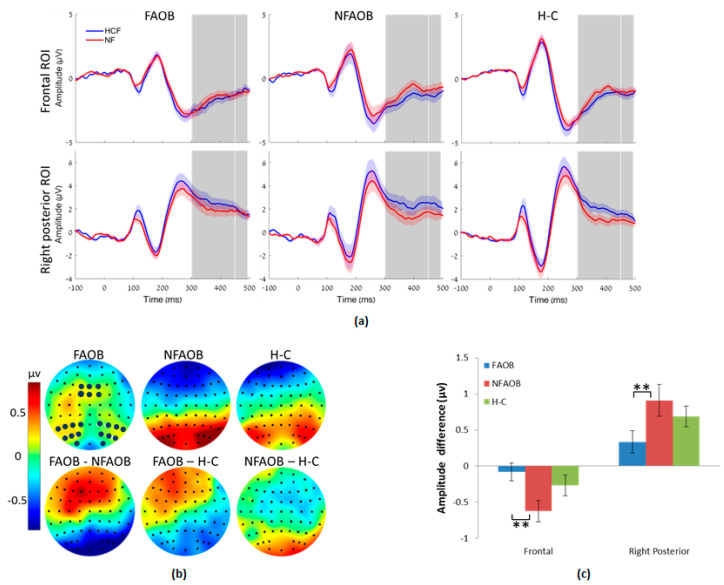
Brain potentials in response to food cues. (**a**) Event related potential curves in the FAOB, NFAOB and H-C groups, following HCF compared with NF images, in the frontal and right posterior ROI (no group differences in the left posterior ROI, hence not shown). Curve’s shades mark SEM; TOIs (LPPa, LPPb) are marked in gray. (**b**) Topographic plots of mean group amplitudes (upper row) and group contrasts (lower row) during the LPPb. Electrodes of the different ROI are marked in dark blue (**c**) Mean and SEM of the same in the frontal and right posterior ROI. ** *p* ≤ 0.01. FAOB: overweight and obese with food addiction. NFAOB: overweight and obese without food addiction. H-C: healthy controls. HCF: high-calorie food. NF: nonfood. ROI: region of interest. SEM: standard error of mean. TOI: times of interest. LPP: late positive potential.

Nonparametrical permutation tests indicated differences between the three groups in brain potentials in response to food cues. There was an amplitude change induced by the food cues (HCF vs. NF), in the LPPb but not in the LPPa, in the frontal (F = 4.09 _(*n* = 52)_, *p* = 0.02) and right occipital (F = 3.78 _(*n* = 52)_, *p* = 0.02) but not in the left occipital ROI (F = 1.40 _(*n* = 52)_, *p* = 0.26) (Figure 4). Post-hoc contrasts revealed that the FAOB group had a lower food cue-induced amplitude change in the LPPb compared with the NFAOB group, in the frontal (*t* = 2.84 _(*n* = 37)_, *p* = 0.0058) and right posterior (*t* = 2.62 _(*n* = 37)_, *p* = 0.0013) ROI. Neither the FAOB nor the NFAOB groups statistically differed from the H-C group. 

**Figure 5:**
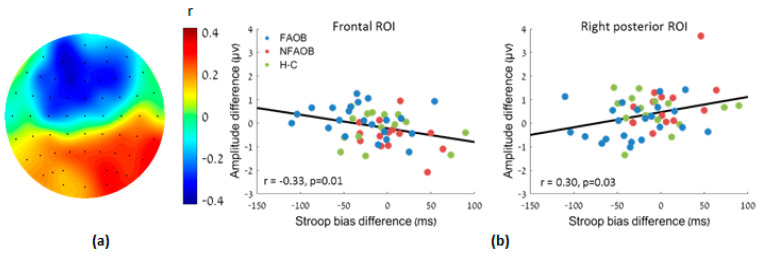
Association between the amplitude change and the Stroop bias change following HCF compared to NF images, for the participants altogether. (**a**) A topographic plot of the linear correlation magnitude between the food-cue induced LPPb amplitude and the Stroop bias changes. (**b**) Scatter plots of the same in the frontal and right posterior ROI. HCF: high-calorie food. NF: nonfood. LPP: late positive potential. ROI: region of interest.

Correlational analysis between the food cue-induced differences in both the Stroop bias and the LPPb amplitude revealed a negative correlation in the frontal ROI (*r*_(*n* = 51)_ = −0.33, *p* = 0.02) and a positive correlation in the right posterior ROI (*r*_(*n* = 51)_ = 0.30, *p* = 0.03; Figure 5). Therefore, the food cue-induced amplitude change was linearly associated with the Stroop bias change following HCF compared to NF images.

## 4. Discussion

In the current study, we aimed to investigate psychobiological indices of food addiction in overweight and obesity. The results provide novel and unique neurobehavioral and psycho-cognitive markers characterizing FA versus no FA in overweight and obese participants. Our hypotheses were partially confirmed; during rest, the FAOB group showed greater left-brain asymmetry than that of the NFAOB group. This neurobiological signature is in line with approach motivation tendencies [82], indicating greater vulnerability to approach a motivationally salient cue relevant to the addictive condition [83]. The greater left-brain asymmetry at rest in the FAOB group may indicate a neuro-marker of repeatedly and compulsively approaching highly rewarding food, similarly to those observed in substance addiction [36]. Conversely, the greater right-brain dominance in the NFAOB group in comparison with the FAOB and (to a lesser extent) the H-C groups, may protect them from developing FA symptoms. The lack of addictive features in the NFAOB group may be reflected in their right frontal asymmetry. An extensive body of research points to inhibitory control deficits in obesity, which is associated with impulsivity and a lack of delayed discounting of food-related reward [84,85]. To overcome impulsivity toward food reward, the NFAOB may have developed heightened compensatory mechanisms in their right brain hemisphere, which is absent in healthy adults and the FAOB group, the latter who may be lacking the capacity to consistently control their food intake. 

Our findings are in line with past research indicating left PFC asymmetry in chronic overeaters [31], in adults with high hedonic hunger (i.e., a drive for a food reward in the absence of hunger) [86], and in obesity [87]. In the current study, we also found left-brain asymmetry in the occipital ROI, and a general trend of reduced left alpha asymmetry in the whole hemisphere, possibly indicating a widespread left hemispheric dominance in the FAOB group, extending to brain areas in the sensory association cortex [77]. Specifically, brain responses with robust asymmetry to the left parietal and occipital brain areas may be related to strategic and tactical aspects of goal pursuit [88], such as reward valuation and integration [77,89], as well as hedonic valuation of food [77]. A widespread asymmetry that includes temporal and occipital electrodes has been observed in healthy individuals [76] and in psychiatric patients [90], but this is the first study that shows this electrophysiological marker in food addiction. Therefore, additional studies are needed to help uncover the significance of these findings. 

In the Food Stroop task, participants viewed pictures of highly rewarding food, as well as nonfood items, before the Stroop word assignment. We hypothesized that the FAOB participants will show greater cue-reactivity in response to highly rewarding food cues, reflected in greater Stroop bias and heightened ERP responses, than that of NFAOB and H-C participants. This is based on previous literature indicating greater AB in the Food Stroop task in obese compared with lean participants [91,92]. Our results refuted previous findings; at earlier stages of cognitive processing (300–450 ms following picture presentation), the HCF pictures elicited heightened emotional reaction similarly in all groups. Thereafter, during the LPPb (at 450–495 ms) the FAOB group seems to have inhibited their emotional response to the HCF cues, differently from the NFAOB group, who displayed clear electrophysiological difference in response to HCF versus NF images. This neurobiological difference between the groups was behaviorally reflected in their differential performance on the Stroop word assignment, which started immediately thereafter. Indeed, in the Food Stroop task, the FAOB group showed a lower Stroop bias following images of HCF, suggesting that the lack of electrophysiological response to HCF images may result from an inhibitory process. The negative correlation between the neuronal response to HCF vs. NF images and the performance during the Stroop task further implies an inhibitory process of affective response, starting at the neuronal level and reducing attention bias on the Stroop task. The positive correlation between the occipital response to HCF vs. NF images and the performance during the Stroop task implies that increased sensory response to these images (without prefrontal inhibition) may induce the increased attention bias on the Stroop task. 

The LPP component is commonly found in obesity research, and it indicates selective and motivated attention to rewarding cues [93], specifically highly rewarding food [94]. Greater LPP component response in addicted vs. non-addicted individuals, following a visual presentation of a cue associated with the addiction, has been shown in cannabis use and may be a neurobiological marker of addiction [36]. High LPP has also been found in response to an acute stressor, when cortisol levels are high [78], implying vigilant attention to a threat. In our sample, the strong inhibition of emotional-motivational reaction to the HCF cues in the FAOB group may point to the hypervigilance-avoidance hypothesis [95,96]. Research is pointing to an interaction between emotional valence and executive control demands in tasks involving attention and cognitive interference [44,97]. Attentional avoidance in a state of emotional vigilance has been observed in dieters who attempt to attentionally avoid pictures of the food they desire [64,98], and in addicted patients exposed to the stimuli they are trying to abstain from [99]. Accordingly, the HCF images in our study may have elicited in the FAOB group a strong affective response. At the early stages of information processing, the FAOB group, similarly to the other two groups, showed an initial heightened emotional response to the appetitive cues, which was thereafter extensively inhibited in the FAOB group, possibly when experiencing hypervigilance with triggers of an emotionally-laden problematic behavior associated with their condition. 

The hypervigilance-avoidance hypothesis has been shown in adults with social phobia, who respond faster in a Stroop paradigm with images of socially challenging situations, following an anxiety-inducing task [100,101]. Similarly, overeating of highly rewarding food has been postulated to function as a relief from the physical tension associated with hypervigilance [102], suggesting an addictive cycle whereby compulsivity develops to relief from the psychophysiological tension associated with the condition. Following this hypothesis, individuals with overweight or obesity and FA may vigilantly detect highly rewarding food cues in their environment, propelling a negative affect and a negative urgency to impulsively consume that food. They may try to counteract their tendency to approach highly rewarding food (to relieve their hypervigilance) by exercising cognitive avoidance, up to the point where they disinhibit their restraint and lose their control over eating. At the point of disinhibition, consumption of the food may function to relieve the physical and emotional tension associated with generalized or cue-specific hypervigilance [84]. This hypothesis is in line with the theoretical understanding of impulsivity and loss of control of eating seeing in FA [5]. These behaviors and their neurobiological precursors may also be reflected in left-brain asymmetry [33]. 

The FAOB differed from the NFAOB (and the H–C) in both binge-eating (BE) and depressive symptoms and in symptoms of emotional and uncontrollable eating. These results are in-line with our hypotheses and replicate previous research comparing individuals with and without FA [103,104]. BED is related to FA [105], but research about distinctions and similarities between the two conditions is in its infancy. FAOB has been suggested to be an extreme form of BED [6]. However, in our sample, only 13 participants out of the 30 (i.e., 43%) in the FAOB group showed BE symptoms [106]. Moreover, BE symptom scores were not correlated with participants’ performance on the Food Stroop task, nor with brain asymmetry scores or ERPs, and the three groups in our study differed in FA symptoms and BMI even when controlling for binge-eating scores. These are novel and important findings, pointing to FA as a unique clinical construct, characterized by distinct psycho-neurobiological markers, above and beyond BE symptoms. Future studies may employ the parameters addressed in the current study to directly compare two cohorts of overweight/obese adults: one with FA and the other with BE symptoms/BED. 

BE and FA may escalate depressive symptoms [5,104], and there is ample evidence to support the co-occurrence of obesity and depression [2]. In our work, despite greater depressive symptoms in the FAOB group compared with the other two groups, symptoms level did not reach clinical significance but more of a melancholic state [107]. It is possible that the uncontrollable compulsion to eat, low self-esteem [19], self-inefficacy in controlling one’s eating and weight [108], and the impairment in the quality of life in overweight/obesity with FA [109], contributed to greater depressive symptoms in the FAOB group. 

The present study has strengths and limitations. This study is the first to find neurocognitive markers and psycho-behavioral correlations in overweight/obesity with FA, using brain asymmetry indices and ERP in a Food Stroop task. In the present study, participants’ hunger and metabolism were carefully controlled for 24 h prior to, and on the day of, the study, to reduce the chance of confounding variables, such as metabolic hunger [47], biasing the results. Future work may regress these and other potential confounders on the neurocognitive parameters we applied in the current study; this was not performed here and may be a shortcoming of the present work. The present study has a limitation in terms of sample size, particularly in the NFAOB and H-C groups. Moreover, participants’ recruitment in the present study poses several limitations to the generalizability of our findings; we did not recruit participants with the co-presence of obesity and SUD [110], and our study lacks a subgroup of FA who shows lean body mass [111], limiting the conclusions to FA in overweight/ obesity. Moreover, we used the original version of the YFAS, which is based on the DSM-IV, since participants’ recruitment started prior to the publication of the most updated version, the YFAS version 2 [112]. Therefore, we did not distinguish between mild, moderate, and severe FA symptoms in participants’ recruitment. We, therefore, suggest these limitations be addressed in future FA studies. Lastly, our research setting has possibly impacted the participants in the study. Future ecological momentary assessment studies may help examine overweight/obesity with FA in a different, more natural setting, to avoid possible confounding factors of conducting research in the lab. 

## 5. Conclusions

Our study uniquely demonstrated that overweight and obese adults with FA show markers of left-brain asymmetry at rest, relative to overweight/obese adults without FA. In addition, the overweight/obese participants with FA show markers of a hypervigilant inhibition of emotional reaction to food triggers that may elicit excessive cravings, evident in a lower LPPb response to HCF images and reduced AB in a Food Stroop task. Our results are in line with psychobiological markers of SUD and behavioral addiction, and they introduce novel understandings of overweight/obesity with FA. Neurocognitive training and neuro-modulatory treatment, such as transcranial magnetic stimulation (TMS), may help rebalance hemispheric symmetry in obesity with FA. Future studies may also address potential therapies to help individuals with FA cope better with environmental stimuli relevant to their condition. 

## Figures and Tables

**Table 1:** Demographic and clinical data of the participants in the study.

Variable	FAOB (M ± SE)	NFAOB (M ± SE)	H-C (M ± SE)	*p* Value
**Age**	39.05 (13.01)	37.56 (12.9)	34 (6.38)	0.34
**Gender (M; F)**	7; 23	6; 10	9; 9	0.24
**Education**	14.3 (0.33)	15.13 (0.52)	15.07 (0.44)	0.27
**BMI**	34.54 (0.76)	34.29 (1.17)	22.98 (0.38)	<0.0001 ^#§
**VAS (hunger)**	4.81 (0.2)	5.5 (0.19)	5.06 (0.28)	0.11
**YFAS-S**	5.71 (0.26)	3.28 (0.54)	1.38 (0.21)	<0.0001 ^#*§
**TFEQ-EE**	9.42 (0.55)	7.75 (0.6)	5.56 (0.45)	<0.0001 ^#*
**TFEQ-UE**	27.96 (0.81)	24.94 (1.18)	16.78 (1.06)	<0.0001 ^#*
**TFEQ-CR**	13.27 (0.62)	13.72 (0.97)	15.31 (1.1)	0.22
**BDI**	10.46 (1.34)	5.07 (1.19)	2.88 (0.9)	<0.0001 #*
**BE**	6.73 (1.19)	1.09 (0.45)	0.07 (0.05)	<0.0001 #*
**PANAS**	17.87 (7.93)	21.73 (8.35)	17.12 (7.25)	0.21

^ NFAOB vs. H-C (*p* < 0.05); # FAOB vs. H-C (*p* < 0.05); * FAOB vs. NFAOB (*p* < 0.05); **§** Controlling for BE. FAOB: overweight and obese with food addiction. NFAOB: overweight and obese without food addiction. H-C: healthy controls.

## Appendix A

**Table A1:** Examples of YFAS items and scoring [45].

Item	Response Categories					Scoring		Criterion
**In the past 12 months:**	**0**	**1**	**2**	**3**	**4**	**0**	**1**	
1. I find that when I start eating certain foods, I end up eating much more than planned.	never	once a month	2–4 times a month	2–3 times a week	4 or more times or daily	0–3	4	Substance taken in larger amount and for longer period than intended
2. I find myself continuing to consume certain foods even though I am no longer hungry.	never	once a month	2–4 times a month	2–3 times a week	4 or more times or daily	0–3	4	Substance taken in larger amount and for longer period than intended
3. I eat to the point where I feel physically ill.	never	once a month	2–4 times a month	2–3 times a week	4 or more times or daily	0–2	3–4	Substance taken in larger amount and for longer period than intended
4. Not eating certain types of food or cutting down on certain types of food is something I worry about.	never	once a month	2–4 times a month	2–3 times a week	4 or more times or daily	0–3	4	Persistent desire or repeated unsuccessful attempt to quit
5. I spend a lot of time feeling sluggish or fatigued from overeating.	never	once a month	2–4 times a month	2–3 times a week	4 or more times or daily	0–2	3–4	Much time/activity to obtain, use, recover
6. I find myself constantly eating certain foods throughout the day.	never	once a month	2–4 times a month	2–3 times a week	4 or more times or daily	0–3	4	Much time/activity to obtain, use, recover
7. I find that when certain foods are not available, I will go out of my way to obtain them. For example, I will drive to the store to purchase certain foods even though I have other options available to me at home.	never	once a month	2–4 times a month	2–3 times a week	4 or more times or daily	0–2	3–4	Much time/activity to obtain, use, recover

**Table A2:** Number of participants included in the analysis.

	FAOB	NFAOB	H-C	Reasons for Drop-out
Stroop Reaction Time	30	15	16	Incomplete recording of RT (1) Failed to record covariate scores (4)
EEG at rest	18	15	16	Insufficient data quality
Event Related Potentials	22	15	15	Insufficient data quality
Food Stroop—ERP correlations	22	14	15	Insufficient data quality

FAOB: overweight and obese with food addiction. NFAOB: overweight and obese without food addiction. H-C: healthy controls. RT: reaction time. ERP: event-related potentials.

## Appendix B

EDE-Q-I questionnaire’s questions assessing BE symptoms [55].

“13. Over the past 28 days, how many times have you eaten what other people would regard as an unusually large amount of food (given the circumstances)?

14. On how many of these times did you have a sense of having lost control over your eating (at the time that you were eating)?

15. Over the past 28 days, how many DAYS have such episodes of overeating occurred (i.e., you have eaten an unusually large amount of food and have had a sense of loss of control at the time)?”
